# Advances in the role of SWI/SNF complexes in tumours

**DOI:** 10.1111/jcmm.17709

**Published:** 2023-03-08

**Authors:** Ziwei Li, Jiumei Zhao, Yu Tang

**Affiliations:** ^1^ Chongqing Health Center for Women and Children Women and Children's Hospital of Chongqing Medical University Chongqing China; ^2^ Chongqing Nanchuan District People's Hospital Chongqing China; ^3^ The Third Affiliated Hospital of Kunming Medical University, Yunnan Cancer Hospital Kunming China; ^4^ Department of Genetics Zunyi Medical University Guizhou China

**Keywords:** diagnosis, molecular mechanism, SWI/SNF complex, treatment, tumour

## Abstract

Cancer development is a complex process involving both genetic and epigenetic changes. The SWI/SNF (switch/sucrose non‐fermentable) chromatin remodelling complex, one of the most studied ATP‐dependent complexes, plays an important role in coordinating chromatin structural stability, gene expression and post‐translational modifications. The SWI/SNF complex can be classified into BAF, PBAF and GBAF according to their constituent subunits. Cancer genome sequencing studies have shown a high incidence of mutations in genes encoding subunits of the SWI/SNF chromatin remodelling complex, with abnormalities in one or more of these genes present in nearly 25% of all cancers, which indicating that stabilizing normal expression of genes encoding subunits in the SWI/SNF complex may prevent tumorigenesis. In this paper, we will review the relationship between the SWI/SNF complex and some clinical tumours and its mechanism of action. The aim is to provide a theoretical basis to guide the diagnosis and treatment of tumours caused by mutations or inactivation of one or more genes encoding subunits of the SWI/SNF complex in the clinical setting.

## 1 INTRODUCTION

Cancer has been one of the most significant challenge for humanity, with the potential to be the most deadliest disease globally. In China, where cancer is the leading cause of death, with an estimated 4.3 million new cancer cases and 2.9 million new cancer deaths in 2018. While the cancer incidence rate is lower compared to the United States and the United Kingdom, the mortality rate is 30% and 40% higher respectively.^1^ The increasing trend of cancer incidence year by year suggests that studying the mechanisms of cancer development will help to reduce the risk of cancer. SWI/SNF is a chromatin remodelling complex, which was first discovered in S. cerevisiae and Drosophila, and in recent years, its structure and function have been reported consistently in humans. Its structures in yeast, drosophila and human are shown in Table 1, and the subunits of the same colour are highly conserved.^2^, ^3^, ^4^, ^5^, ^6^ In mammals, it can be broadly classified into three categories based on the specific subunits of its composition: PBAF, dominated by PBRM1, ARID2 and BRD7; BAF, dominated by ARID1A/ARID1B and DPF2; GLTSCR1/GLTSCR1L and BRD9‐dominated GBAF.^2^ The SWI/SNF is a multiprotein complex essential for regulating eukaryotic gene expression.^3^ With about 4 × 10^13^–6 × 10^13^ cells in the human body and 10^4^–10^5^ DNA damages occurring per cell per day, timely function of SWI/SNF complex is very important. If it fails to function in time, it will lead to intracellular genomic instability, affecting its normal physiological processes, such as transcription and replication, then potentially cause premature aging and even tumorigenesis.^4^, ^5^ Thus, it can be seen that the SWI/SNF complex plays a critical role in the normal physiological activities of humans, which ensures the proper expression of genes in cells, including biological processes such as replication, transcription, translation and post‐translational modifications, ultimately affecting the normal physiological state of human body. In this paper, we will discuss the relationship between the SWI/SNF complex and the SWI/SNF complex in terms of its composition and function, the tumours caused by common mutation types, the main oncogenic mechanisms and the therapeutic approaches for mutations in the subunits of the SWI/SNF complex. We hope to provide new ideas for the study of tumours caused by mutations in the SWI/SNF complex.

**TABLE 1:** SWI/SNF class remodeler subunits in S.cerevisiae, Drosophila and humans.

S. cerevisiae		Drosophila		Human		
ySWI/SNF	RSC	BAP	PBAP	BAF	PBAF	GBAF
SWI2/SNF2	STH1	BRM		SMARCA2/hBRM SMARCA4/BRG1/BAF190		
ARP9		BAP55		ACTL6A/BAF53A ACTL6B/BAF53B		
ARP7		ACT5C		ACTB		
SWI3	RSC8	MOR		SMARCC1/BAF155		
				SMARCC2/BAF170		
SWP73/SNF12	RSC6	BAP60		SMARCD1/BAF60A		
				SMARCD3/BAF60C		
				SMARCD2/BAF60B		
SNF5	SFH1	SNR1		SMARCB1/SNF5/BAF47/INI1		
		BCL7		BCL7A BCL7B BCL7C		
		BAP111		SMARCE1/BAF57		
	RSC1 RSC2 RSC4		PBRM		PBRM1/BAF180	
	RSC9		BAP170		ARID2/BAF200	
			SAYP		PHF10/BAF45A	
			BRD7		BRD7	
SWI1		OSA/ eyelid		ARID1A/BAF250A ARID1B/BAF250B		
		D4 TTH		DPF1‐3/BAF45B‐D		
		SS18		SS18 SS18L1		SS18 SS18L1
		CG11873				GLTSCR1 GLTSCR1L
		CG7154				BRD9
RTT102						
SNF6						
SNF11						
TAF14						
SWP82						
	RSC3					
	RSC30					
	RSC7					
	RSC14					
	RSC58					
	HTL1					

## 2 STRUCTURE AND FUNCTION OF SWI/SNF

### 2.1 Structure of SWI/SNF

**FIGURE 1:**
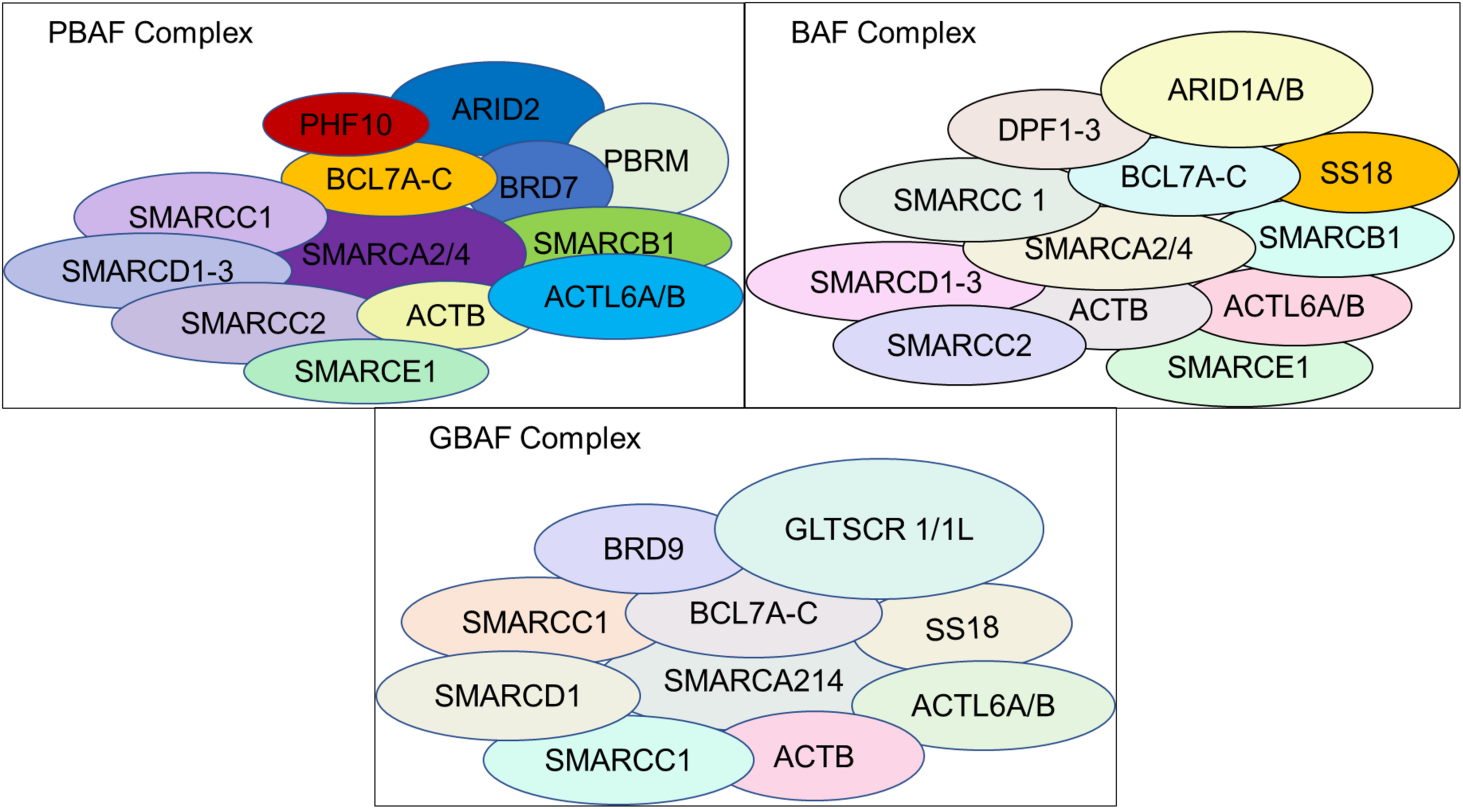
Types of SWI/SNF chromatin remodelling complexes and their structures.

The SWI/SNF complex is a chromatin remodelling complex that plays a key regulatory role in the development of many tumours or diseases.^3^ It was first identified in Saccharomyces cerevisiae by screening for genes involved in encoding HO nucleases, which were found to be required for mating‐type switching (SWI),^6^ to affect the expression of SUC2 transactivases required for sucrose fermentation (SNF),^7^ and to encode a number of proteins, including SNF2ATPase, which were shown to reside in a common complex, hence the name SWI/SNF complex.^8^ SWI/SNFs can now be broadly classified into three categories based on the specific subunits they comprise: BAF, PBAF, and GBAF/ncBAF, with BAF and PBAF is highly conserved in eukaryotes,^2^, ^9^ and GBAF/ncBAF is a new type only recently discovered in mammals.^10^ Their structures are shown in (Figure 1). Among them, SMARCA2/4, SMARCC1, SMARCCD1, ACTB, ACTL6A/B, BCL7A‐C are the subunits common to the three types of SWI/SNF complexes, and the other subunits are the constituent components of each. As shown in the figure, the SWI/SNF complex is composed of proteins encoded by multiple genes and therefore must play an important role in the normal physiological activities of cells.

### 2.2 Functions of SWI/SNF

The main molecular function of the SWI/SNF complex is chromatin remodelling by disrupting nucleosomes. This requires transcriptional regulators and other proteins to recruit SWI/SNF complexes to DNA regions, which alter the stability of nucleosomes upon binding to these proteins, and then transcriptional regulators bind to the exposed DNA to repress or activate gene expression.^11^ In yeast, approximately 6% of genes are estimated to be regulated by the SWI/SNF complex.^12^ In contrast, mammalian SWI/SNF complexes exist in multiple forms and are characterized by distinct constitutive subunits, including BRM/SMARCA2 or BRG1/SMARCA4 (ATPases that hydrolyse ATP) and a group of other proteins called BRG1/BRM‐associated factors (BAFS) that are required for binding to DNA or proteins.^13^ In human somatic cells, three of these subunits (INI1/SMARCB1, BAF155/SMARCC1 and BAF170/SMARCC2) are called ‘core subunits’ as they are required for the chromatin remodelling activity of the ATP‐dependent SWI/SNF complex in complex with BRM or BRG1‐related factors.^13^ It has been suggested that the heterogeneity of SWI/SNF complex subunits in higher eukaryotes can maintain the changing cellular requirements and specific functions of differentiated cells.^14^ Furthermore, genome‐wide sequencing analysis has revealed that subunits of the SWI/SNF complex in human somatic cells are closely associated not only with promoter binding but also with interactions between other regions regulating DNA expression, such as enhancers and DNA replication initiation sites, and in addition, subunits of the SWI/SNF complex have been found to bind to many proteins that make up the nucleoskeleton and cytoskeleton,^15^ suggesting that the function of the SWI/SNF complex in human cells may have a broader function than purely transcriptional regulation.

The biological effects of the SWI/SNF complex and its subunits were also discovered to cover a broad range of cellular functions. BRG1 and another SWI/SNF complex subunit, INI1, for example, promote DNA repair in response to DNA damage,^16^ hBRM affects selective splicing of genes in human cells,^16^ and genome‐wide analysis of human cell samples has revealed that SWI/SNF complex subunits, such as BRG1, affect the expression levels of approximately 4.8%–20% of genes.^17^, ^18^ Subunits of the SWI/SNF complex have been shown to regulate gene expression levels in a variety of cellular signalling pathways, including signal transduction, cell cycle, apoptosis, cell adhesion, cell morphology, DNA repair and cellular stress responses.^19^ These findings suggest that the SWI/SNF complex is not only involved in the transcriptional regulation of genes, but also plays a critical role in connection with the normal physiological functions of human cells. Mutations or loss of genes encoding subunits of the SWI/SNF complex may cause disruptions in cellular physiological functions, leading to imbalances in the human organism's homeostatic and even tumorigenesis.

## 3 MUTATIONS IN SWI/SNF SUBUNITS CAUSE HUMAN TUMOURS

Tumorigenesis involves many complex molecular genetic and epigenetic events, and SWI/SNF, as a multi‐protein complex, contains many subunits, one or more of which encode constitutive subunits of gene development. Mutations or loss of section can lead to changes in its function and induce tumours. For example, as can be seen in Table 2, common mutations in SWI/SNF subunits induce the development of certain tumours. In addition, related studies have reported that ARID2, SMARCC1, SMARCD1‐3 and other subunits have a low mutation probability in tumours, which is manifested as delection.^20^, ^21^, ^22^, ^23^, ^24^, ^25^, ^26^, ^27^, ^28^

**TABLE 2:** SWI/SNF common mutations in cancer.

Mutant genes	Name of cancer	Type of mutations	Reference
PBRM1	RCC	Truncating mutations (34%), nonsense, missense and frameshift mutations	^32^
	PDAC	Deletion	^33^
	BC	Truncating mutations	^36^
SNF5	RMS	Homozygous deletion, nonsense, missense and frameshift mutations	^37^
	SCUD	Translocations and homozygous deletion of 22q11.2	^38^
	EMC	Frameshift and homozygous deletion	^39^
	USTS	Homozygous deletion and intragenic mutation	^40^
	ES	Homozygous deletion	^41^
	PDCs	Loss of 22q11.2	^42^
ARID1A	OCCC	Truncating mutations	43, 44
	EC	Truncating mutations	43, 44
	RCC	Homozygous deletions and heterozygous missense mutations	^32^
	ICD	Truncating mutations	^45^
	LC	Intergenic deletion	^46^
	BC	Genomic rearrangement	^46^
BRG1	NSCLC	Homozygous truncating mutations and missense mutations	^47^
	LC	Missense, insertion and nonsense mutations	48, 49, 50, 51
	ICD	Missense mutations	^45^
	PDA	Truncating mutations and missense mutations	^52^
	BC	Truncating mutations and missense mutations	^52^
	PC	Truncating mutations and missense mutations	^52^
	RT	Truncating mutations	^53^
BRD7	BC	Genomic loss on chromosome arm 16q. Reduced expression in 20% of primary tumours	^20^

Of all SWI/SNF subunit genes, BAF250A/ARID1A is the most commonly mutated gene in human cancers: the gene is mutated in a large proportion of ovarian clear cell cancers (~45%–57%), ~30% of endometrioid cancers, a small proportion of bladder cancers and other tumours.^29^, ^30^ This represents that of all the subunits of SWI/SNF, ARID1A may be particularly important in its function. PBRM1, a target subunit of PBAF, is a common oncogene with a 40% mutation rate in renal cell carcinoma, and our previous study found that deletion of PBRM1 could activate the AKT pathway and glycolytic pathway and thus affect the function of ccRCC cells.^31^ Furthermore, mutations in PBRM1 are also associated with pancreatic cancer (PDAC), advanced gastric cancer (GC), cervical cancer (CCA) and other tumour formation related.^32^, ^33^, ^34^, ^35^, ^36^ BRD7, SMARCC2, ARID2, ACTL6A/B, SMARCC1, SMARCD1/2/3 are other key subunits of SWI/SNF, and recent studies have reported that mutations in these subunits cause gastric cancer (GC), colon cancer (CRC), hepatocellular carcinoma (HCC), head and neck squamous carcinoma (HSNC), breast cancer (BRCA) and other tumours.^20^, ^21^, ^22^, ^23^, ^24^, ^25^, ^26^, ^27^, ^28^, ^37^, ^38^, ^39^, ^40^, ^41^, ^42^, ^43^, ^44^, ^45^, ^46^, ^47^, ^48^, ^49^, ^50^, ^51^, ^52^, ^53^ In addition, genes encoding tumour‐associated subunits of the SWI/SNF complex, such as BAF57/SMARCE1, are heterozygous for mutations or completely lost in familial spinal meningioma cases.^31^ Some of these tumours contain mutations in more than one subunit, such as renal cell carcinoma, breast cancer, and colon cancer, suggesting that the development of a cancer may be caused by mutations in one or more subunits of the SWI/SNF complex. Does the mutation of several subunits increase the risk of cancer or progress cancer? This issue deserves a closer examination and verification.

In addition to the above subunits that are often mutated, one subunit mutation found in a subunit of the SWI/SNF complex is a germline mutation, which predisposes individuals to cancer, namely the core subunit INI1, whose mutations have been detected in rhabdomyosarcoma, an aggressive childhood cancer characterized by tumours in sites such as the kidney and soft tissues,^54^ and in addition, its double allele inactivation causes familial nerve sheath tumour disease and meningioma,^55^, ^56^ and the loss of INI1 protein has been found to cause epithelioid sarcoma.^57^ Interestingly, with INI1 than that of the other two core subunits encoding the SWI/SNF complex (BAF155/SMARCC1 and BAF170/SMARCC2), which are rarely mutated in human cancers,^58^ suggesting that mutations in IN1 increase the risk of cancer in individuals.

Mutations in genes encoding subunits of the SWI/SNF complex are also present in other tumours, including gliomas, medulloblastomas, and squamous cell carcinomas,^3^, ^58^, ^59^ where mutations in SWI/SNF complex subunit genes include mutations in BRG1 and, to a lesser extent hBRM.^58^ BRG1 is the gene encoding the SWI/SNF complex subunit that was found to be associated with human cancers after BAF250A/ARID1A Mutations in subunits of the SNF complex have been detected in BRG1 mutations in hereditary and sporadic high‐glucose ovarian small cell carcinoma, a rare, undifferentiated and aggressive cancer characterized by early onset, although uncommon, in which germline mutations and somatic inactivation of BRG1 have been observed, while no associated germline mutations have been reported for BRM.^60^ This study demonstrates that it can play a more significant role in cancer than BRM, as confirmed by statistical data on hereditary cancers.^58^ In summary, mutations in a single subunit of SWI/SNF or co‐mutations in multiple subunits may increase the risk of cancer development, suggesting that SWI/SNF has an extremely important role in tumour formation and may play a role as an oncogenic molecule.

## 4 MAJOR ONCOGENIC MECHANISMS OF SWI/SNF COMPLEXES

### 4.1 Effect of SWI/SNF on gene transcription

Mutations or inactivation of genes encoding subunits of the SWI/SNF complex are found in approximately 20% of cancers.^61^ The most widely studied area of the SWI/SNF complex is the transcriptional regulation of genes, which can maintain cellular functional homeostasis by regulating the expression of certain specific genes. However, the process of transcriptional regulation requires not only the involvement of ATPases, but also other subunits of the SWI/SNF complex that play important roles by directly stimulating or repressing certain transcriptional regulators.^62^ For example, ARID1A/B and SNF5 can interact with MYC proteins to regulate the expression of their target genes and can also affect the expression of the MYC gene itself,^62^ which is known to be an oncoprotein; therefore, inactivation of subunits of the SWI/SNF complex may cause an increase in MYC expression and promote tumorigenesis. Similarly, the subunits BRG1 and BRM of the SWI/SNF complex can bind to the oncogene RB1 and enhance the expression activity of RB1, thus suppressing the expression of E2F family genes and arresting cell growth in the G1 phase,^63^ inhibiting tumour progression.

### 4.2 Effect of SWI/SNF on cancer‐related pathways

Besides its ability to affect transcriptional regulation, recent studies have shown that SWI/SNF can also influence the tumour development process by inhibiting cancer‐related pathways. For example, SWI/SNF can interact with the ARIDA subunit through the key protein YAP/TAZ binding in the HIPPO signalling pathway reduces YAP/TAZ expression and inhibits tumour cell proliferation as well as stemness,^64^ SWI/SNF subunit BRD9 can mediate changes in androgen signalling pathway to affect prostate cancer progression.^65^ In addition, the SWI/SNF complex can directly regulate the Notch signalling pathway and affect the tumour development process.^66^ Our experimental results show that PBRM1, the target subunit of SWI/SNF, can inhibit AKT signalling pathway and glycolytic pathway to suppress the proliferation and migration of clear renal cell carcinoma cells.^31^ As a result, many critical pathways in tumours are regulated by SWI/SNF, and disrupting the homeostasis of these pathways can exacerbate the formation of tumours.

The above studies suggest that SWI/SNF is an oncogenic complex in tumours, which may affect the transcriptional process of genes through its chromatin remodelling effect, and then some of its subunits can interact with cancer‐related proteins to regulate the expression changes of reciprocal proteins or their downstream genes, in addition, it can also exert oncogenic effects by regulating certain critical pathways in tumours, thus controlling the tumour development and the process of tumour development.

## 5 TRANSLATION OF SWI/SNF MUTATIONS IN THE CLINIC

Most subunits of the SWI/SNF complex have been shown to have oncogenic effects, and their inactivation promotes tumorigenesis and has a strong correlation with patient prognosis and difficulty of treatment.^67^ Therefore, drugs and treatments for cancers caused by inactivation of these subunits are slowly being investigated. For example, Tatiana Shorstova et al.^68^ found that low doses of bromodomain and ectodomain inhibitors (BETi) had significant antiproliferative effects in vitro and in vivo on invasive ovarian and lung cancer models lacking the SMARCA4 and SMARCA2 subunits, key components of the SWI/SNF chromatin remodelling complex.

Shuai Wu et al.^15^ found that inactivation of ARID1A, the most common mutant subunit of the SWI/SNF complex in tumours, caused resistance to EZH2 inhibitors, but was sensitive to BCL2 inhibitors, and that the combination of BCL2 and EZH2 inhibitors gave good therapeutic results in a mouse ovarian cancer model. The main mechanism of action of EZH2 inhibitors is to inhibit its H3K27 methylation enzyme activity. Kim KH et al.^69^ found that the ARID1A mutation caused cancer EZH2‐dependent proliferation with only a small portion relying on the catalytic effect of the methylation enzyme of EZH2 and most relying on the interaction of EZH2 with other proteins. Therefore, the treatment of EZH2‐dependent tumours after ARID1A mutation requires the inhibition of its interaction with proteins in addition to the inhibition of the methylesterase activity of EZH2. These studies provide new ideas for the treatment of tumours, and EZH2‐dependent tumours caused by ARID1A inactivation can be treated with appropriate selection of BCL2 inhibitors.

Several other molecular biological therapies have emerged for the treatment of ovarian cancer with mutations in the SWI/SNF complex subunits ARID1A and SMARCA4, mainly divided into the following categories: (1) epigenetic synthetic lethality; (2) activation of the pro‐apoptotic effect of wild‐type p53; (3) checkpoint blockade immunotherapy; (4) DNA damage signalling inhibitors; (5) cellular kinase signalling pathways.^70^ These therapies target either the enhanced proliferative capacity of ovarian cancer cells caused by mutations in ARID1A and SMARCA4 or directly promote apoptosis of the tumour cells to achieve therapeutic goals.

PBRM1, another component of the SWI/SNF complex, is found mutated in 41% of renal clear cell carcinomas, and sunitinib, a common clinical kidney cancer treatment, has no significant effect on PBRM1‐mutated renal clear cell carcinomas, but it is sensitive to anti‐VEGF drugs.^71^, ^72^ In addition, a new EZH2 inhibitor, L501‐1669, has a^73^, which suggests that clear renal cell carcinoma cells can be eliminated by drugs.

**FIGURE 2:**
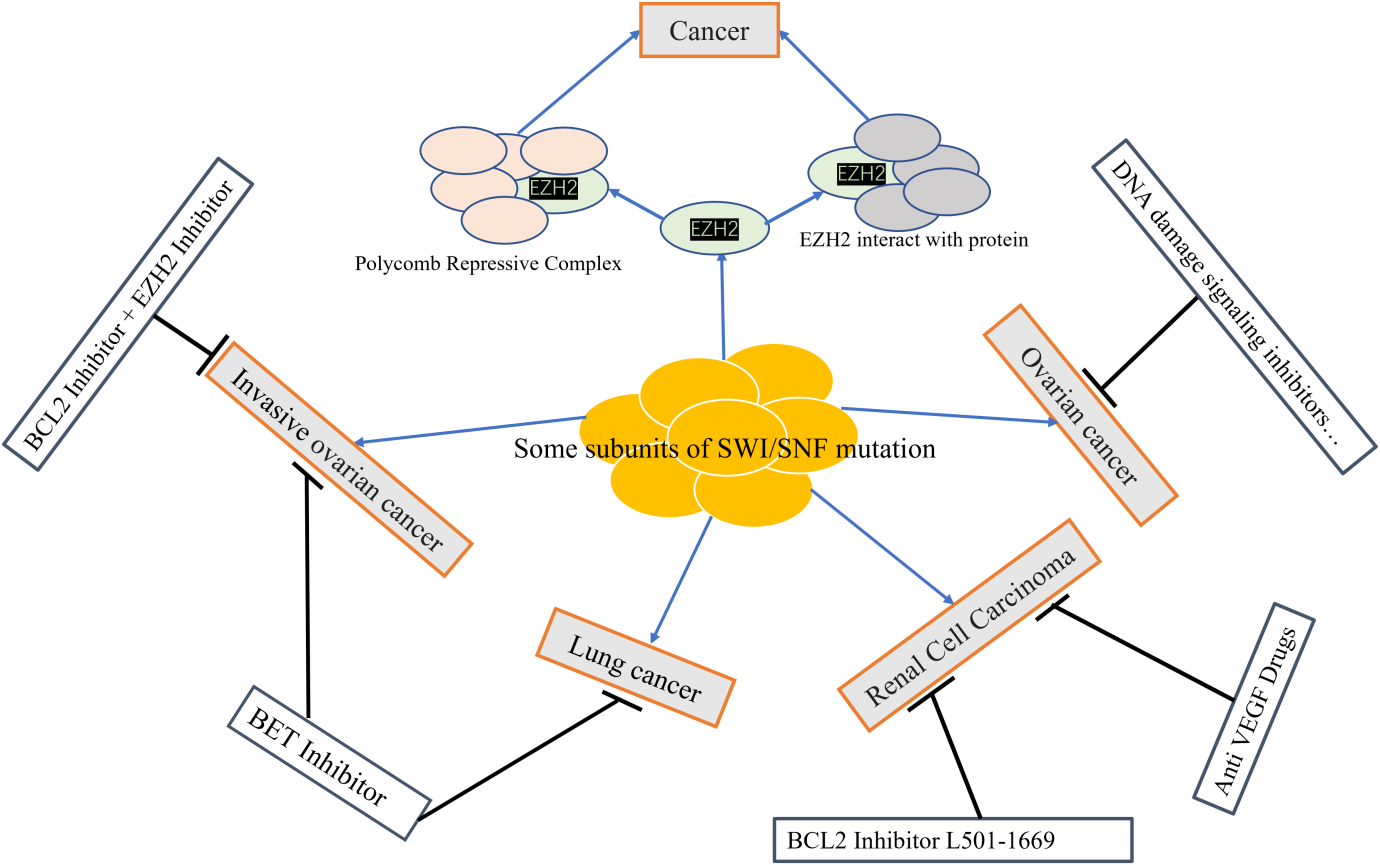
Tumours caused by SWI/SNF partial subunit mutations and their treatment.

The above studies suggest that tumours caused by mutations in one or more subunits of the SWI/SNF complex can be suppressed by specific clinical agents (Figure 2). At present, most of the treatment methods are applied to the treatment of ovarian cancer, and there are few reports on targeted therapy in other tumours. So targeted studies on the mutation of SWI/SNF complex subunits in these cancers can undoubtedly increase our knowledge of tumour development, provide effective evidence for the design of targeted molecular drugs, and even lay a solid foundation for the adjuvant diagnosis and treatment of clinical tumours, with the ultimate goal of enhancing the possibility of tumour prevention and cure.

## 6 CONCLUSION

Cancer is by far the most lethal disease other than atherosclerosis, and it is a serious threat to human health. Nowadays, the correlation between the SWI/SNF chromatin remodelling complex and tumours has been increasingly studied, but most of the studies are still limited to the mutation of some subunits of the SWI/SNF complex to induce tumours, which only slightly reveals the mechanism of action, but not the specific site of action. Some drugs have been found to treat tumours with partial subunit inactivation of the SWI/SNF complex, but these drugs are targeted to patients with distinct clinical features and are not targeted to the SWI/SNF complex. Nowadays, only mutations in ARID1A have been more comprehensively studied in ovarian cancer, explaining not only that their mutations cause an increase in MYC expression and affect tumour progression, but also that therapies targeting ARID1A mutations have emerged in the clinic. Currently, PBRM1 has been studied more frequently and more comprehensively mechanistically, but only in clear renal cell carcinoma. In addition, the SWI/SNF chromatin remodelling complex is a multi‐protein complex, and the relevance of mutations in the remaining subunits to tumours is still poorly studied, as these subunits are mutated in 20% of tumours, suggesting that they are also likely to play an important role in tumour development. The SWI/SNF complex is known to be associated with gene transcription, but there are few reports on which genes it regulates, and the activation of tumour‐associated pathways is also associated with the SWI/SNF complex. However, there are no reports on molecular targeting drugs. Therefore, an in‐depth study of the mechanism of action of SWI/SNF complex in tumours can provide sufficient theoretical basis for drug development, clinical adjuvant diagnosis and treatment of tumours caused by its mutations.

## AUTHOR CONTRIBUTIONS

**Li Zi Wei:** Data curation (equal); funding acquisition (equal); software (equal). **Zhao Jiu Mei:** Data curation (equal); investigation (equal); resources (equal); software (equal). **Yu Tang:** Data curation (equal); investigation (equal); writing – original draft (lead); writing – review and editing (lead).

## FUNDING INFORMATION

This work was supported by the grants from the Scientific Research project of Education Department of Yunnan Province [2023Y0787].

## CONFLICT OF INTEREST STATEMENT

The authors confirm that there are no conflicts of interest.

## Data Availability

The references are derived from PubMed, which is completely open and can be downloaded freely.
